# Identification of ferroptosis-related genes in the progress of NASH

**DOI:** 10.3389/fendo.2023.1184280

**Published:** 2023-05-25

**Authors:** Linwei He, Jianming Wang, Baihua Tao, Ruolan Zhu, Changbing Li, Bo Ning

**Affiliations:** ^1^ Department of Gastroenterology, The Second Affiliated Hospital, Chongqing Medical University, Chongqing, China; ^2^ Biology Science Institutes, Chongqing Medical University, Chongqing, China; ^3^ Department of Gastroenterology, The People’s Hospital of Fengjie County, Chongqing, China

**Keywords:** non-alcoholic steatohepatitis, *CDKN1A*, *SIRT1*, ferroptosis, bioinformatics

## Abstract

**Background:**

Non-alcoholic steatohepatitis (NASH) is becoming more widespread, and some similarities exist between its etiology and ferroptosis. However, there are limited investigations on which ferroptosis-related genes (FRGs) are regulated in NASH and how to regulate them. We screened and validated the pivotal genes linked to ferroptosis in NASH to comprehend the function of ferroptosis in the development of NASH.

**Methods:**

Two mRNA expression data were obtained from the Gene Expression Omnibus (GEO) as the training set and validation set respectively. FRGs were downloaded from FerrDb. The candidate genes were obtained from the intersection between differentially expressed genes (DEGs) and FRGs, and further analyzed using the Gene Ontology (GO) and Kyoto Encyclopedia of Genes and Genomes (KEGG). The hub genes were identified by the protein-protein interaction (PPI) network and Cytoscape. Then, FRGs closely related to the severity of NASH were identified and further confirmed using the validation set and mouse models. Ultimately, based on these genes, a diagnostic model was established to differentiate NASH from normal tissues using another data set from GEO.

**Results:**

A total of 327 FRGs in NASH were acquired and subjected to GSEA. And 42 candidate genes were attained by overlapping the 585 FRGs with 2823 DEGs, and enrichment analysis revealed that these genes were primarily engaged in the fatty acid metabolic, inflammatory response, and oxidative stress. A total of 10 hub genes (*PTGS2、IL1B、IL6、NQO1、ZFP36、SIRT1、ATF3、CDKN1A、EGR1、NOX4*) were then screened by PPI network. The association between the expression of 10 hub genes and the progress of NASH was subsequently evaluated by a training set and verified by a validation set and mouse models. *CDKN1A* was up-regulated along with the development of NASH while *SIRT1* was negatively correlated with the course of the disease. And the diagnostic model based on *CDKN1A* and *SIRT1* successfully distinguished NASH from normal samples.

**Conclusion:**

In summary, our findings provide a new approach for the diagnosis, prognosis, and treatment of NASH based on FRGs, while advancing our understanding of ferroptosis in NASH.

## 1 Introduction

The prevalence of non-alcoholic fatty liver disease(NAFLD) worldwide is estimated at 32.4%, varying by region (1). Non-alcoholic steatohepatitis (NASH) and non-alcoholic fatty liver (NAFL) are the components of NAFLD, which is the liver phenotype of metabolic syndrome. Steatosis, necrotizing inflammation, and ballooning of hepatocytes are features of NASH (2). If NASH is left untreated, fibrosis may develop, leading to cirrhosis and its complications, and culminating in the end-stage liver disease (3). Compared to patients with NAFL, individuals with NASH experience a faster rate of fibrosis progression, and the appearance of fibrosis, particularly advanced fibrosis (stages 3 and 4), is a significant predictive factor for outcomes involving the liver and overall survival (4–6). Hepatocyte-macrophage-hepatic stellate cells (HSCs) crosstalk is one of the most significant cellular networks, and it controls the development of fibrosis rather than a single-cell type. The cell-cell network that promotes fibrosis is triggered by hepatocyte death (7), which can take many different forms such as apoptosis (8), necroptosis (9), necrosis, pyroptosis (10), and ferroptosis (11). Emerging studies suggest that multiple types of hepatocyte death may drive fibrosis, even though it was once believed that apoptosis was the most predominant cell death in NASH (8). These several types of hepatocyte death may be controlled by overlapping molecular processes and can act as “backups” for one another (12).

Ferroptosis, which distinguishes it from apoptosis, unregulated necrosis, and necroptosis, was defined as an iron-dependent, regulated, and distinctive form of cell death brought by lipid peroxidation (13). Accumulating evidence has shown that ferroptosis plays a role in many pathological processes including iron-overload disease (14), sedaghatian-type spondylometaphyseal dysplasia (SSMD) (15), organ injury (16), and tumor suppression (17–19), in addition to some physiological processes including immune functions (20), development, and aging (21). It is a widely held view that individuals with NAFLD/NASH frequently experience iron overload (22–24). Malondialdehyde and 4-hydroxynonenal, the secondary products of lipid peroxidation, have been shown in several studies to be utilized as oxidative stress indicators in NASH patients (25), and Vitamin E, an antioxidant that suppresses lipid peroxidation, can diminish serum aminopherase in NASH patients (26). Additionally, the increasing iron was found in the liver and serum of mice models feeding MCD-diet to mimic NASH. Surprisingly, ferroptosis inhibitors could attenuate liver damage, fibrosis, and inflammation brought on by the MCD-diet (27). In light of the fact that there is unmet therapy for NASH, it is essential to keep looking into the genes linked to ferroptosis in NASH as they could provide novel insight for prognosis and treatment.

In this study, differentially expressed genes (DEGs) were identified between NASH patients and the control group using GSE135251. Candidate genes were acquired by intersecting DEGs and FRGs. 10 hub genes were further obtained through the PPI network and Cytoscape. Among the 10 hub genes, 7 were highly correlated with the NASH fibrosis stage in GSE135251, 2 were correlated with the NAFLD fibrosis stage in GSE213621 and 5 were correlated with the occurrence of NASH in the mouse models. Finally, we confirmed the overlapping genes of these three parts as the final key genes to establish a diagnostic model. Overall, these findings provide an innovative idea for NASH molecular markers and therapeutic targets while enhancing our understanding of the ferroptosis mechanism in NASH.

## 2 Materials and methods

### 2.1 Data collection

**Figure 1:**
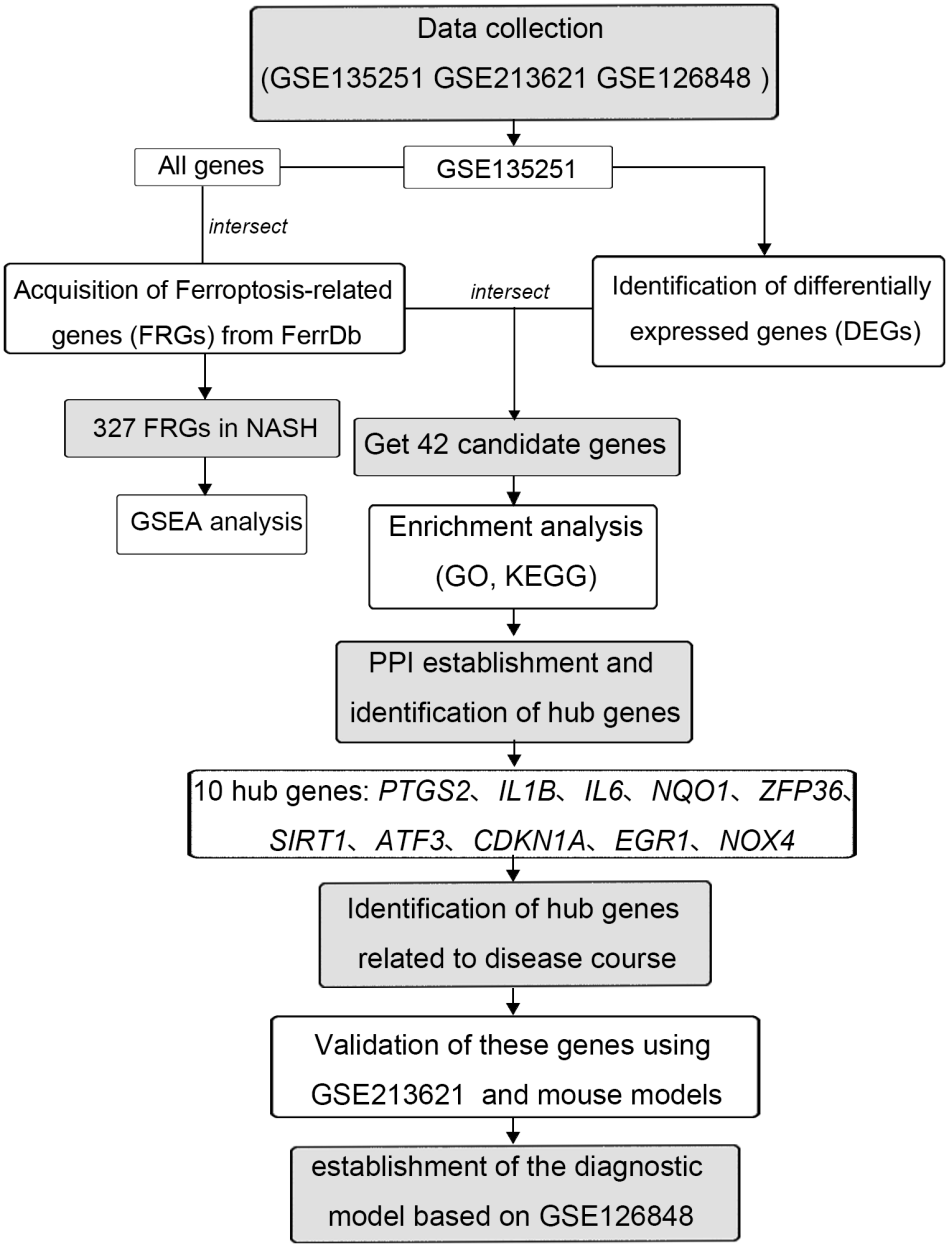
The flowchart of this study.

The expression profiles of high throughput mRNA sequencing dataset GSE135251 based on the platforms of the GPL18573 (28), GSE213621 based on the platforms of the GPL16791, and GSE126848 based on the platforms of the GPL18673 were downloaded from the GEO database in NCBI (http://www.ncbi.nlm.nih.gov/gds/) (29) (**Table 1**). 155 NASH liver tissue samples and 10 healthy liver tissue samples make up the training set GSE135251. As a validation set, GSE126848 including 299 samples of NAFLD-affected liver tissue and 69 samples of normal liver tissue was used. Introduce another dataset GSE126848 that includes 16 NASH tissue samples and 26 normal tissue samples to establish a diagnostic model. For additional study, FRGs were downloaded from FerrDb, an artificial ferroptosis database for managing and identifying ferroptosis-related markers, regulatory factors, and disorders (30). **Figure 1** displays a flowchart of the investigation.

**Table 1:** Characteristics of the GEO datasets.

Datasets		Component	Type	Tissue	Species
GSE135251	training set	NASH:155 Normal:10	Expression profiling by high throughput sequencing	Liver	Homo sapiens
GSE213621	validation set	NAFLD:299 Normal:69	Expression profiling by high throughput sequencing	Liver	Homo sapiens
GSE126848	diagnostic model cohort	NASH:16 Normal:26	Expression profiling by high throughput sequencing	Liver	Homo sapiens

### 2.2 Identification of differentially expressed genes

Using the DESeq2 package of R, Genes that met the threshold requirements of |log2FC| >1 and adjusted p-value< 0.05 were selected and served as DEGs (31). The principal components analysis(PCA) plot, expression heatmap, and volcano plot of the DEGs were created using the “ggbiplot”, “pheatmap” and “ggplot2” packages via R software.

### 2.3 Gene set enrichment analysis

To preliminarily explore the potential mechanism of ferroptosis in NASH, 327 genes, acquired by crossing 38094 genes in GSE135251 with FRGs, were used for Gene Set Enrichment Analysis (GSEA) via the ClusterProfiler package of R software. The number of permutations is 1000, NES>1, p-value<0.05, and q-value<0.25 are set as the critical values for significant enrichment. The corresponding Venn diagram is drawn by the R package “VennDiagram”.

### 2.4 Gene ontology and Kyoto Encyclopedia of Genes and Genomes

The candidate genes were obtained by crossing the DEGs and FRGs, and were displayed by the R package “VennDiagram”. ClueGO, a Cytoscape plug-in, visualized the enrichment pathway of GO and KEGG database to explore the probable roles of candidate genes in NASH. The p-value<0.05 was deemed significant enrichment. And the visual style is set to “group”, the Kappa score threshold was 0.4 (32).

### 2.5 Protein-protein interaction network construction

Protein interactions and linkages were structured using the STRING online database (https://string-db.org/). After that, Cytoscape (https://cytoscape.org) was used to visualize the PPI network. Finally, the Cytoscape software’s cytoHubba plug-in was used to screen the hub genes (33).

### 2.6 Validation of hub genes using the validation dataset

The box plot is used to demonstrate the association between the expression of hub genes and the fibrosis stage of the training set. Then, the hub genes’ expression information, extracted from the expression matrix of data set GSE213621, was taken as validation data. The difference in gene expression between several groups was analyzed using Student’s t-test. The result was shown in a violin plot drawn by R package “ggplot2”.

### 2.7 Animal experiments

Twenty C57BL/6 J mice (male, weight 18-21 g, seven weeks old) were purchased from the ENSIWEIER (Chongqing, China). All mice were housed in an independent ventilation cage (IVC) at a 12-h light/dark cycle. Following a week of acclimation, mice were randomly assigned to one of four groups: MCD-diet (XTMCD, Feed-science) fed for four or six weeks; control diet fed for four or six weeks. After the conclusion of the treatments, the mice were sacrificed by cervical dislocation. Subsequently, the liver tissues were photographed, quickly frozen with liquid nitrogen, and maintained at −80 °C for additional research. All animal protocols were reviewed and approved by the ethics committee of Chongqing Medical University.

### 2.8 Histological analysis

A part of each mouse’s liver tissue was fixed in 4% paraformaldehyde for a minimum of 24 hours at room temperature. Afterward, the dehydrated samples were embedded in paraffin or immediately embedded in an OCT medium and stored at -20 °C. Paraffifin-embedded samples, sectioned into 4 μm, were stained with hematoxylin-eosin (H&E). Frozen sections were sectioned into 8 μm and stained with Oil red O solution. Images were acquired using the Eclipse E100 microscope (Nikon, Japan). According to criteria outlined by a well-validated grading system, the NAS score, which ranged from 0 to 8, was calculated by adding the scores of steatosis (0–3), lobular inflammation (0–3), and hepatocyte ballooning (0–2). NAS score ≥ 5 can determine the success of mouse modeling. Two qualified pathologists scored the samples in a blind fashion.

### 2.9 Quantitative Real-Time PCR (qRT-PCR)

Reverse transcription was carried out using a PrimeScript RT Reagent Kit (Takara, Japan) under the manufacturer’s instructions after total RNA from liver tissues was extracted using Trizol reagent (Takara, Japan). SYBR Green Premix Ex Taq (Takara, Japan) was used for quantitative real-time PCR on a Light Cycler 480 (Roche, Switzerland), and results were analyzed using the 2^−ΔΔCt^ technique with β-actin serving as an internal control for normalization. The primer sequences used in qRT-PCR were shown in **Table 2**.

**Table 2:** Primers were used for RT-PCR analysis.

Gene Symbol	Species	Forward Primer	Reverse Primer
*Il6*	Mouse	CTGCAAGAGACTTCCATCCAG	AGTGGTATAGACAGGTCTGTTGG
*Il1b*	Mouse	GAAATGCCACCTTTTGACAGTG	TGGATGCTCTCATCAGGACAG
*Ptgs2*	Mouse	TTCCAATCCATGTCAAAACCGT	AGTCCGGGTACAGTCACACTT
*Egr1*	Mouse	TCGGCTCCTTTCCTCACTCA	CTCATAGGGTTGTTCGCTCGG
*Atf3*	Mouse	GAGGATTTTGCTAACCTGACACC	TTGACGGTAACTGACTCCAGC
*Sirt1*	Mouse	GAGCTGGGGTTTCTGTCTCC	CTGCAACCTGCTCCAAGGTA
*Cdkn1a*	Mouse	CCTGGTGATGTCCGACCTG	CCATGAGCGCATCGCAATC
*Nqo1*	Mouse	AGGATGGGAGGTACTCGAATC	TGCTAGAGATGACTCGGAAGG
*Zfp36*	Mouse	TCTCTGCCATCTACGAGAGCC	TCCTCCGAGGGATTCGGTTC
*Nox4*	Mouse	GAAGGGGTTAAACACCTCTGC	ATGCTCTGCTTAAACACAATCCT
*β-actin*	Mouse	GGCTGTATTCCCCTCCATCG	CCAGTTGGTAACAATGCCATGT

### 2.10 Molecular diagnostic model

To further verify whether FRGs contribute to accurate diagnosis of NASH, a diagnostic model based on the FRGs that were well validated and related to the development of NASH was constructed by using binomial logistic regression analysis. And the diagnostic equation structured by the R language *glm* function was defined as follows:


$$\text{logit}(p)=\ln\frac{p}{1-p}=\beta_{0}+\beta_{1}x_{1}+\beta_{2}x_{2}+\mathrm{...}+\beta_{n}x_{n}$$

*β_n_ *is the correlation coefficient of gene *n*, and *X_n_* is the expression level of gene *n*.

The receiver operating characteristic (ROC) analysis was used to evaluate the diagnostic performance of the diagnostic model.

## 3 Results

### 3.1 Identification of DEGs related to NASH

**Figure 2:**
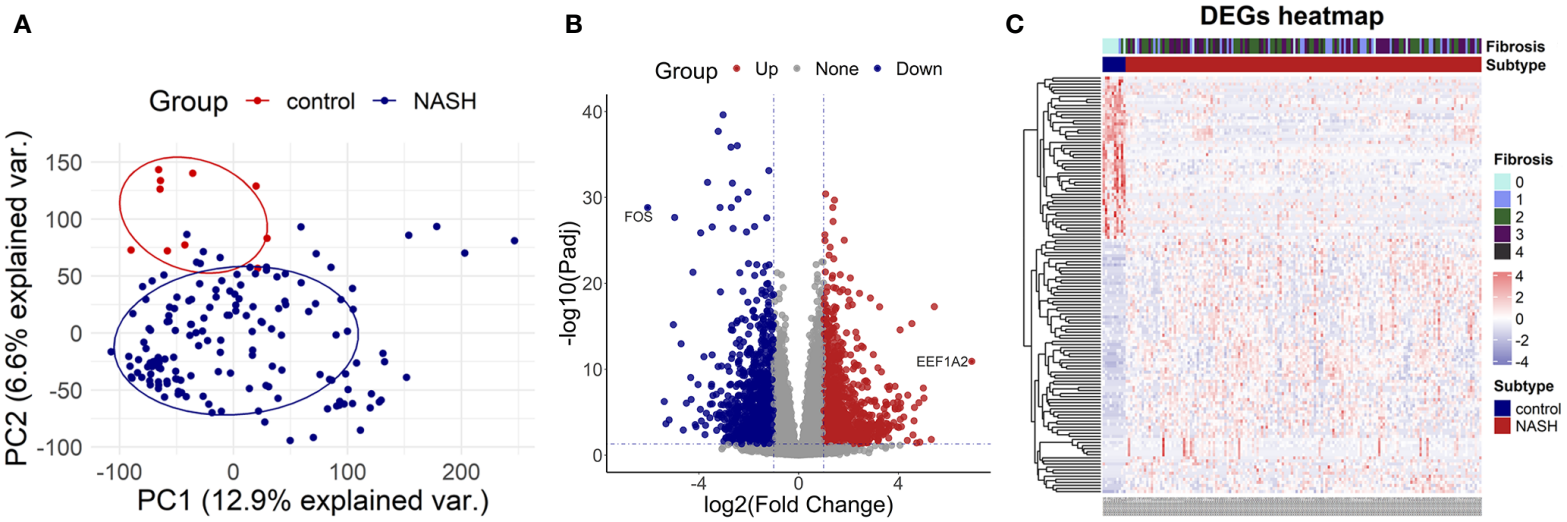
Identification of differentially expressed genes **(A)** the principal components analysis graph (PCA) of dataset GSE135251. **(B)** The volcano map of dataset GSE135251. **(C)** The heatmap of dataset GSE135251.

To investigate the DEGs between the NASH group and the control group, we selected the high throughput sequencing dataset GSE135251 with a total of 38,094 genes to conduct principal components analysis (PCA). The result demonstrated that NASH and the control group were distributed in different regions, suggesting distinct genes expressed between the two groups (**Figure 2A**). Then 2,823 DEGs were identified in total, with 1,290 genes highly up-regulated and 1,533 genes significantly down-regulated in NASH samples compared to healthy samples. (**Figures 2B, C**).

### 3.2 Gene set enrichment study (GSEA)

**Figure 3:**
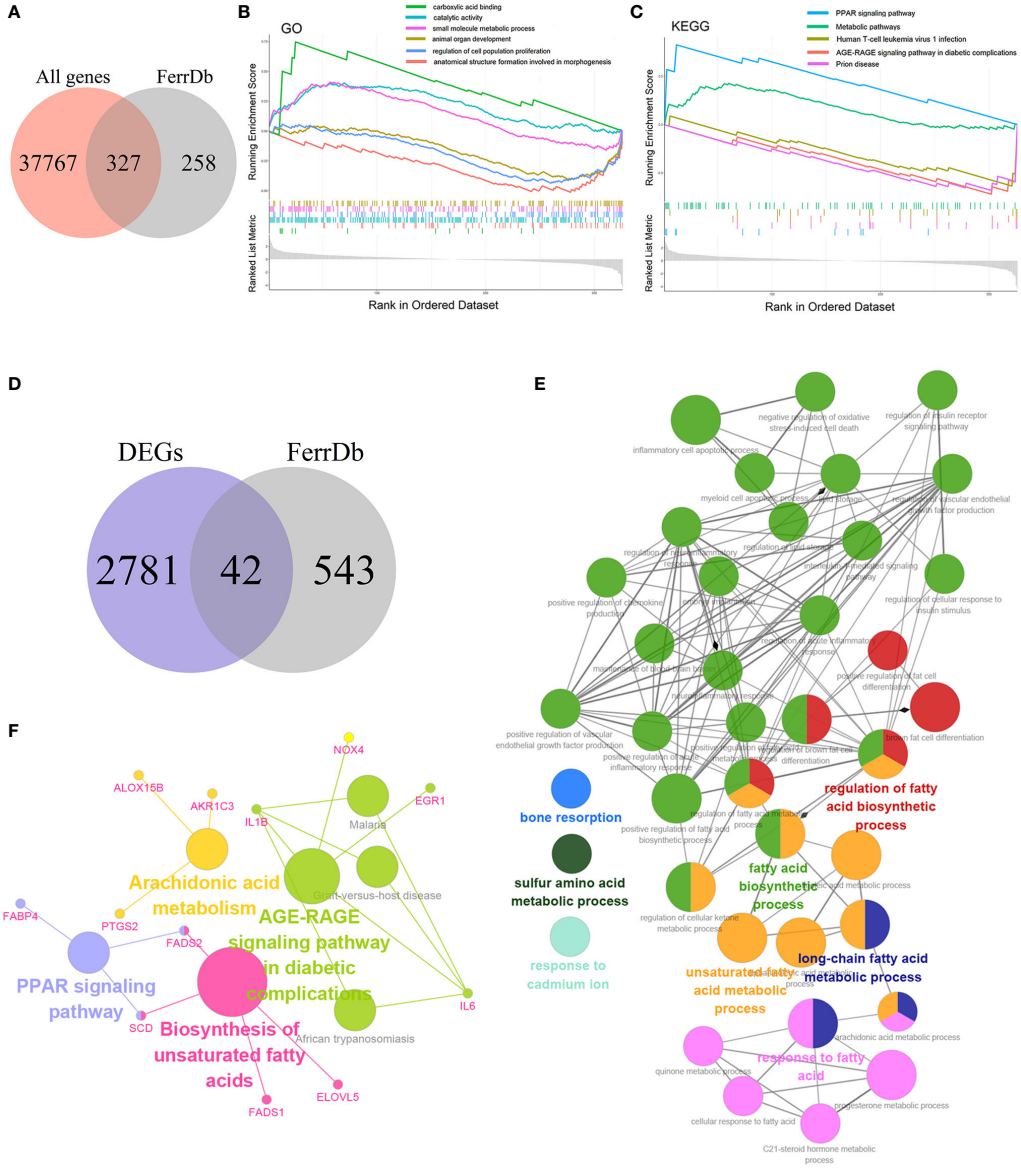
Enrichment analysis **(A)** Venn diagram of the intersection of all genes and FRGs. **(B)** The Gene Set Enrichment Analysis (GSEA) based on GO. **(C)** The Gene Set Enrichment Analysis (GSEA) based on KEGG. **(D)** Venn diagram of the intersection of DEGs and FRGs. **(E)** GO functional annotation of 42 candidate genes. **(F)** KEGG pathway enrichment of 42 candidate genes.

To explore the relationship between ferroptosis and NASH, we acquired 585 genes with high confidence, including drivers, suppressors, and markers for ferroptosis from FerrDb. Then 585 genes were intersected with 38094 genes from GSE135251, and 327 FRGs related to NASH were obtained (**Figure 3A**). The Gene Set Enrichment Study (GSEA) analysis of these 327 genes indicated that the up-regulated FRGs in NASH were involved in carboxylic acid binding, catalytic activity, the small molecule metabolic process, the PPAR signaling pathway, and metabolic pathways, while the down-regulated FRGs were engaged in animal organ development, regulation of cell population proliferation, anatomical structure formation involved in morphogenesis, human T-cell leukemia virus 1 infection, the AGE-RAGE signaling pathway in diabetic complications, and prion disease (**Figures 3B, C**).

### 3.3 Enrichment analysis of candidate genes

The 42 candidate genes were obtained through overlapping 585 genes downloaded from FerrDb with 2,823 DEGs (**Figure 3D**). To identify the function of these genes in NASH, the biological process of GO and KEGG database were utilized and then visualized using the Cytoscape ClueGO bioinformatics tool. The 39 GO terms and 79 connections were acquired respectively and shown in **Figure 3E**, suggesting that the candidate genes mainly involved in fatty acid metabolism (e.g., fatty acid biosynthetic process, unsaturated fatty acid metabolic process, and long-chain fatty acid metabolic process), immune mechanisms (e.g., inflammatory cell apoptotic process, positive regulation chemokine production, and interleukin-1-mediated signaling pathway), and negative regulation of oxidative stress-induced cell death (**Supplementary Table S1**). Additionally, the KEGG enrichment result indicated that the 42 genes were implicated in the PPAR signaling pathway, the biosynthesis of unsaturated fatty acids, the AGE-RAGE signaling pathway in diabetic complications, and the arachidonic acid metabolism (**Figure 3F**).

### 3.4 Protein-protein interaction network establishment and identification of hub genes

**Figure 4:**
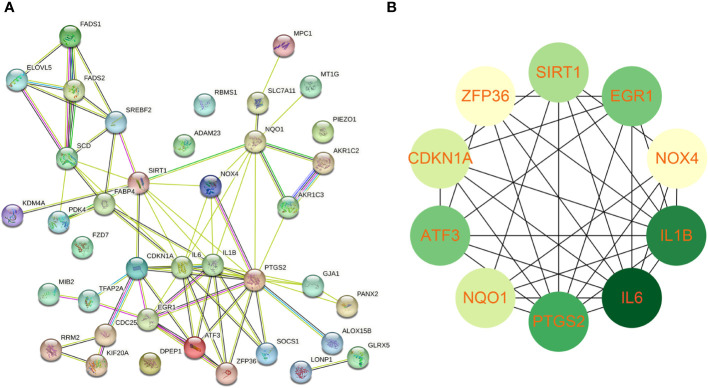
Protein-protein interaction network establishment and identification of hub genes **(A)** protein-protein interaction network of 42 candidate genes. **(B)** The top 10 genes based on the MCC algorithm served as hub genes. The darker the color, the higher the ranking.

**Figure 5:**
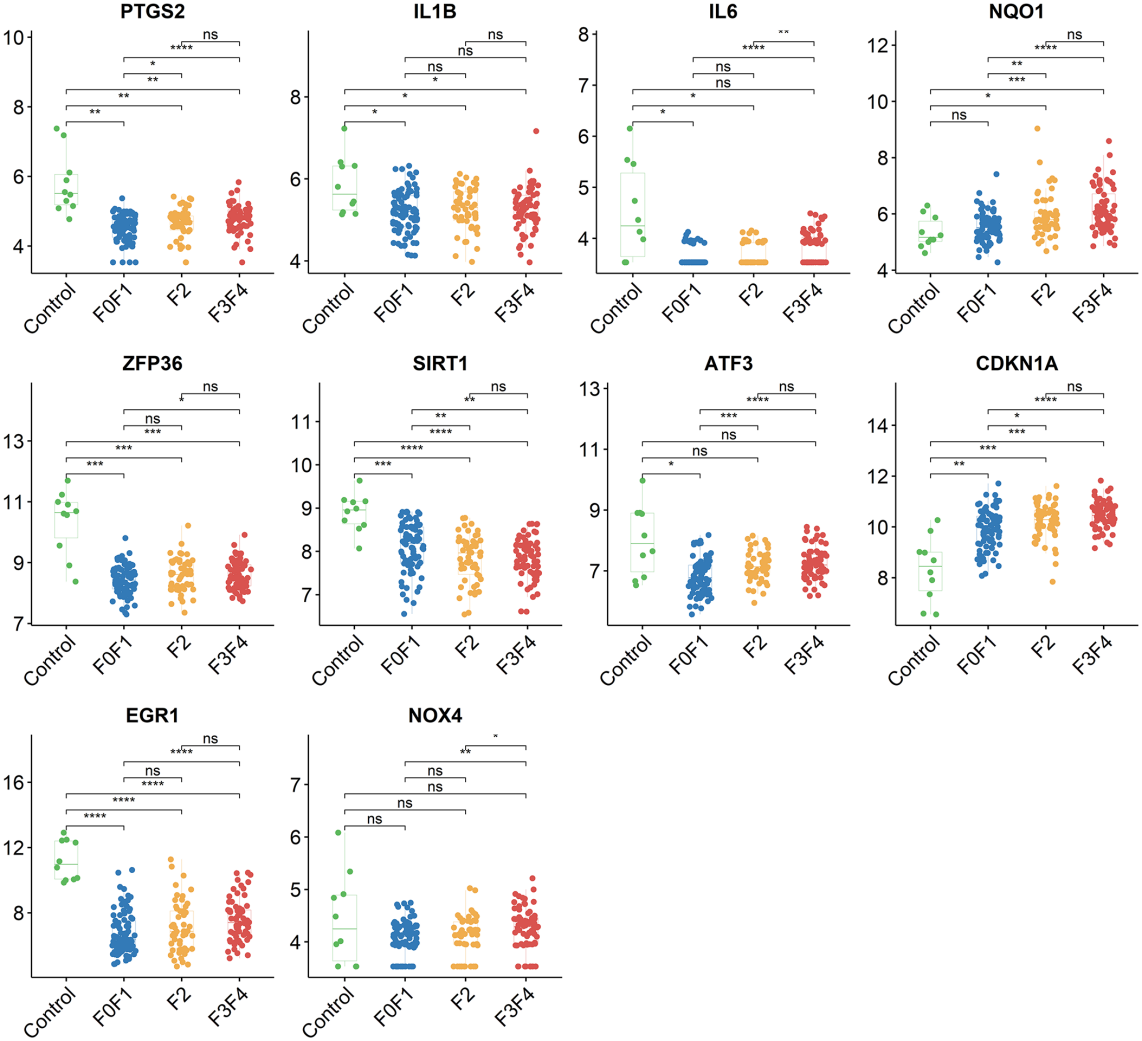
The association between the expression level of 10 hub genes and the fibrosis stage in GSE135251, ns, no significance; *p<0.05, **p<0.01, ***p<0.001, ****p<0.0001.

Using the STRING online database, the PPI network of 42 genes relevant to ferroptosis was created (**Figure 4A**). Subsequently, the top 10 genes based on the MCC algorithm were determined with cytoHubba plug-in of Cytoscape and defined as hub genes including *PTGS2、IL1B、IL6、NQO1、ZFP36、SIRT1、ATF3、CDKN1A、EGR1、NOX4* (**Figure 4B**), the detailed information was illustrated in **Table 3**. Considering that the fibrosis stage is an essential prognostic factor of NASH, we evaluated the association between the expression level of 10 hub genes and the fibrosis stage. The results showed that compared to the control group, *PTGS2*, *ZFP36*, *ATF3*, and *EGR1* were significantly down-regulated in the FOF1 group of NASH patients (p<0.01, 0.001, 0.05, and 0.0001 respectively), but their expression levels presented an upward trend as fibrosis worsened. The expression of *CDKN1A* and *NQO1* were higher than those of the control group and significantly up-regulated with the increase of fibrosis stage. The expression level of *SIRT1* was considerably negatively correlated with the stage of fibrosis in NASH patients (**Figure 5**).

**Table 3:** Top 10 in PPI network ranked by MCC method.

Gene Symbol	Rank	Full Name	Score	Ferroptosis
*IL6*	1	Interleukin 6	402	Driver/Suppressor
*IL1B*	2	Interleukin 1 Beta	400	Driver
*PTGS2*	3	Prostaglandin-Endoperoxide Synthase 2	399	Marker
*EGR1*	4	Early Growth Response 1	242	Driver
*ATF3*	5	Activating Transcription Factor 3	240	Driver
*SIRT1*	6	Sirtuin 1	151	Driver/Suppressor
*CDKN1A*	7	Cyclin Dependent Kinase Inhibitor 1A	147	Suppressor
*NQO1*	8	NAD(P)H Quinone Dehydrogenase 1	126	Suppressor
*ZFP36*	9	ZFP36 Ring Finger Protein	120	Suppressor
*NOX4*	9	NADPH Oxidase 4	120	Drive

### 3.5 Validation of hub genes through the validation set

**Figure 6:**
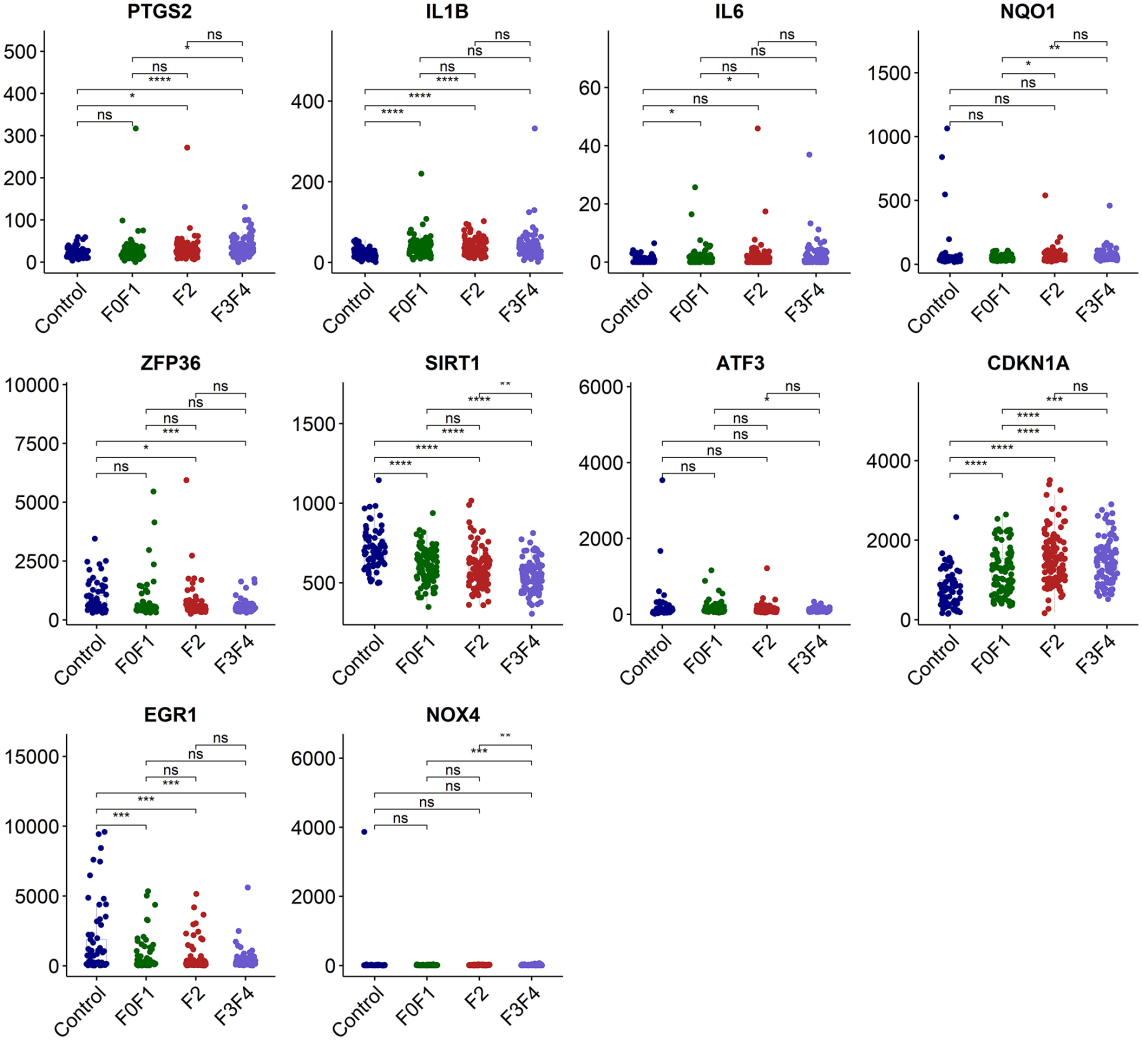
Validation of the hub genes in dataset GSE213621. ns, no significance; *p<0.05, **p<0.01, ***p<0.001, ****p<0.0001.

We employed the data set GSE213621 to further investigate the expression pattern of the hub genes. In the validation set, there was a statistically significant difference in the expression of *IL1B* and *EGR1* between the NAFLD group and the control group (p<0.0001 and 0.001 respectively), but it was weakly correlated with fibrosis staging. *CDKN1A* was significantly up-regulated with the occurrence of NAFLD and the development of fibrosis, while *SIRT1* was significantly down-regulated (**Figure 6**).

### 3.6 The expression level of hub genes in NASH mouse models

**Figure 7:**
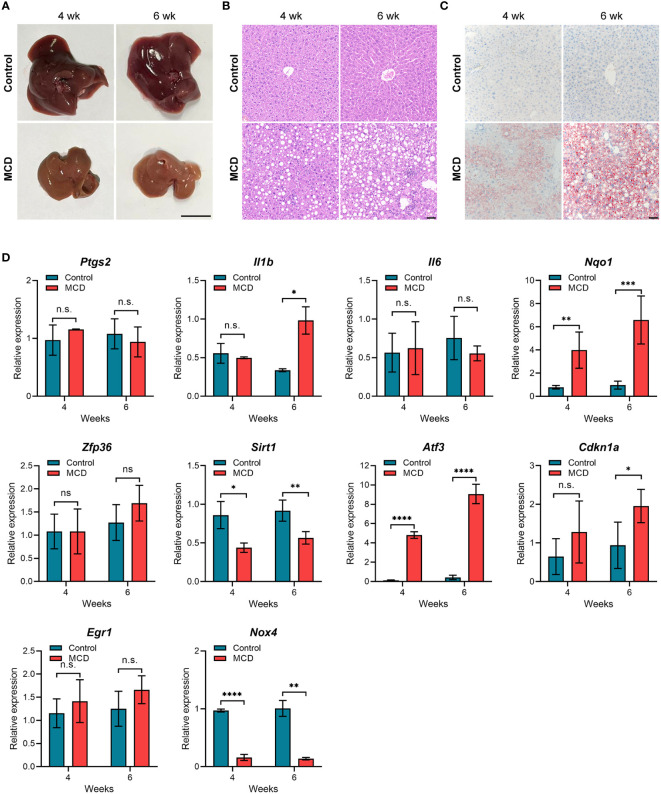
Animal experiment **(A)** Gross morphology of hepatic tissue from control and MCD diet mice (4 or 6 weeks on the indicated diet). Scale bar: 1 cm. **(B)** HE staining of liver sections from mice fed on control or MCD-diet (4 or 6 weeks on the indicated diet). Scale bar: 50 μm. **(C)** Oil Red O stained neutral lipids in the liver from mice fed on the control or MCD-diet(4 or 6 weeks on the indicated diet). Scale bar: 50 μm. **(D)** Determination of hub gene expression in the liver of the control group or MCD-diet mice by RT-PCR (n=5 per group). n.s, no significance; *p<0.05, **p<0.01, ***p<0.001, ****p<0.0001.

To further verify the expression of the hub genes in NASH, we constructed NASH mice models. After 4 weeks of feeding on the MCD-diet, we noticed that mouse liver tissue became pale and faint yellow, which was more pronounced in the 6-week group, indicating fat accumulation in the liver (**Figure 7A**). As expected, the liver tissue of MCD diet-fed for 6 weeks mice showed more extensive steatosis and Inflammatory cell infiltration than those MCD diet-fed for 4 weeks according to histological hepatic sections stained with H&E and Oil-red O (**Figures 7B, C**). To analyze the relevance between the expression of 10 hub genes and the disease progression of NASH, we detected the expression levels of these genes through qPCR. As shown in **Figure 7D**, compared with the control group, *Nqo1*, *Atf3*, and *Cdkn1a* were up-regulated in MCD diet-fed mice, and gradually increased with the aggravation of steatosis and inflammation. Conversely, *Sirt1* and *Nox4* showed a significant decrease in MCD diet-fed mice compared to the control group but were not noticeably related to feeding time. Eventually, we selected the genes that are consistently expressed in GSE135251, GSE213621, and mouse models as our final key genes, namely *CDKN1A* and *SIRT1.*

### 3.7 Establishment of the diagnostic model based on *SIRT1* and *CDKN1A*

**Figure 8:**
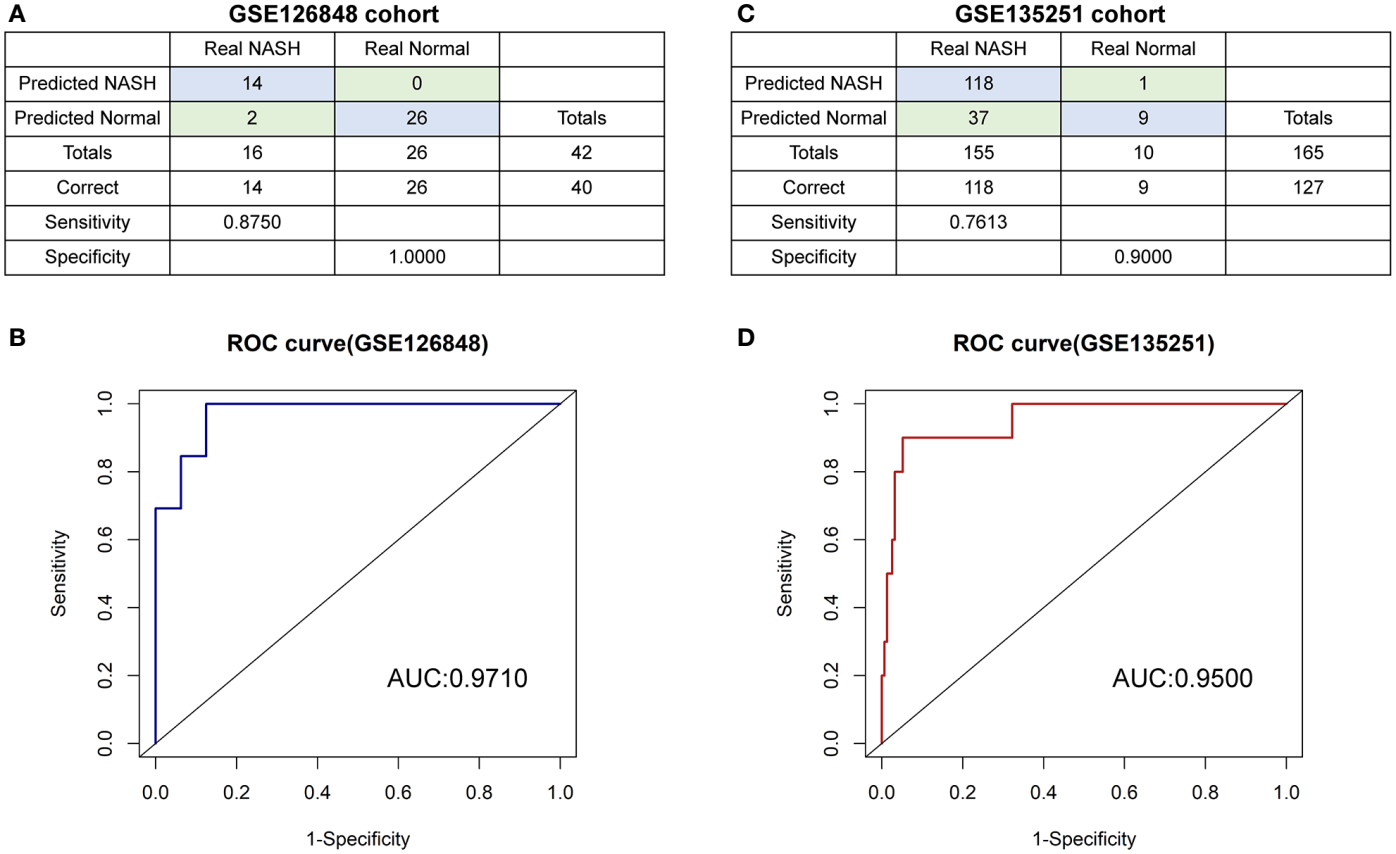
The diagnostic model **(A, B)** the confusion matrix and ROC curve of the diagnostic training cohort (GSE126848). **(C, D)** the confusion matrix and ROC curve of the diagnostic verification cohort (GSE135251).

A diagnostic model based on *SIRT1* and *CDKN1A* was further constructed by using the binomial logistic regression analysis. The diagnostic equation was finally identified as follows: logit (*p*=NASH) =3.729739424 + (-0.027860906×expression level of *SITR1*) + (0.005796107 × expression level of *CDKN1A*). Keeping the expression level of *CDKN1A* unchanged, the Odds Ratio (OR) for the expression level of *SITR1* with one unit increases is 0.9725236 (95%CI 0.9491139-0.9866785). Similarly, the OR for the expression level of *CDKN1A* with one unit increases holding other factors fixed in the model is 1.0058129 (95%CI 1.0011365-1.0134521). It is evident from these results that the low expression of *SIRT1* and the high expression of *CDKN1A* have a higher likelihood of having NASH. In the GSE126848 cohort containing 26 normal samples paired with 16 NASH samples, our diagnostic model achieved a sensitivity of 87.50% and a specificity of 100.00% (**Figure 8A**). After that, applying the diagnostic model to the GSE135251 consisted of 10 normal samples and 155 NASH samples resulting in a sensitivity of 76.13% and a specificity of 90.00% (**Figure 8C**). As shown in ROC analysis (**Figures 8B, D**), the AUCs of our model reached 0.971 and 0.950 in two cohorts respectively, denoting a satisfactory accuracy of prediction.

## 4 Discussion

A significant economic burden was imposed by the prevalence of NASH, which was also accompanied by a rise in the number of patients needing liver transplants due to cirrhosis and end-stage liver disease (34). Regulated cell death is pivotal in governing outcomes of NASH, in which different types of cell death may coexist and transform with each other. Recent investigations have shown that inhibiting apoptosis, necroptosis, or pyroptosis only partially abates liver damage in NASH (10, 35), suggesting that additional kinds of cell death are likely to be to blame for the hepatotoxicity linked to NASH. Ferroptosis, a newly discovered form of regulated cell death, was contributed by 1) the incorporation of activated polyunsaturated fatty acids (PUFAs) into membrane lipids and subsequent peroxidation, 2) the accumulation of reactive oxygen species (ROS), and 3) the Fenton reaction aided by both the labile iron pool and iron-dependent enzymes (16). Several lines of investigations proved that the occurrence of NASH along with the production of lipid peroxides (36, 37), which meets the onset of ferroptosis, suggesting ferroptosis may engage in the progress of NASH. Understanding the process of ferroptosis in NASH may consequently enable the identification of novel prognostic, diagnostic markers, and therapeutic targets and shed light on the pathogenic underpinnings of the disease. In the current study, a total of 42 candidate genes were extracted from the intersection of DEGs acquired from GSE135251 and FRGs from the FerrDb database. As expected, we found that enrichment terms of these candidate genes contain regulation of lipid metabolism, inflammatory response, and oxidative stress, associated with the initial and development of NASH. Furthermore, we established the PPI network of all candidate genes. According to the MCC algorithm in the cytoHubba plug-in of Cytoscape, the top 10 genes were selected as hub genes including *PTGS2、IL1B、IL6、NQO1、ZFP36、SIRT1、ATF3、CDKN1A、EGR1、NOX4.* It has been reported that the fibrosis development of NASH experienced five stages, F0 to F4, representing no fibrosis, sinusoidal fibrosis, sinusoidal and portal fibrosis, bridging fibrosis, and cirrhosis respectively (38). We then deciphered the connection between hub genes and NASH advancement. Our data showed that only *SIRT1* and *CDKN1A* were closely related to the progress of NASH in GSE135251, GSE213621, and mouse models. Additionally, a diagnostic model based on *SIRT1* and *CDKN1A* was built with excellent discrimination in NASH tissues.

*CDKN1A*, also termed p21, is a pivotal regulator of the cell cycle and is engaged in multiple types of cancer, metabolic dysfunctional diseases, and inflammation (39, 40). A high expression level of *CDKN1A* was strongly associated with the exacerbation of NAFLD fibrosis and predicted a poor prognosis in both animal experiments and human NASH samples (41, 42), which was supported by our findings. Recent research indicated that *CDKN1A*, as a transcriptional target of *P53*, was a negative regulator for ferroptosis in cancer cells, which can maintain the glutathione level and reduce lipotoxic ROS (43). The relationship between *CDK1NA* and ferroptosis in NASH was not clear yet, which is worth further exploration. *SIRT1*, a class III histone deacetylase, is NAD+ dependent and regulates metabolism, growth, and transcription in a variety of organs (44). *SIRT1* deficiency can lead to metabolic disorders such as diabetes mellitus, neurodegeneration, and NAFLD (45). Here, we showed that the *SIRT1* level dropped with the progression of NASH using bioinformatics. In mice models, compared with the control group, the expression of *Sirt1* was decreased in the MCD-diet group, while it showed an upward trend with feeding time without statistical significance. We conjecture there may be the following reasons: 1) The duration of MCD-diet feeding was too short to reach a certain degree of disease severity; 2) The expression level of *Sirt1* may be more related to the stage of fibrosis in NASH, but no fibrosis was observed in our models; 3) Differences in gene expression exist between human and mouse. Despite the research on the action of *SIRT1* on ferroptosis in liver disease is limited, it has been indicated that ulinastatin can protect against acetaminophen-induced liver damage by minimizing ferroptosis via activating the SIRT1/NRF2/HO-1 pathway (46). Intriguingly, resveratrol, a potent SIRT1 activator, modulates FOXO1 deacetylation and thus resists iron overload-induced liver tissue damage in iron overload mouse models (47). Furthermore, iron overload aggravates lipid accumulation and oxidative damage induced by a high-fat diet in mice simulating NAFLD, and the activation of *SIRT1* can alleviate this condition (48). We hypothesize that the effect of ferroptosis on the development of NASH may be the comprehensive result of multiple genes acting through multiple pathways. Based on *CDKN1A* and *SIRT1* expression, the diagnostic model constructed was also determined to be able to distinguish NASH patients from normal individuals with high specificity and high sensitivity. To sum up, the FGRs *CDKN1A* and *SIRT1* may regulate the progression of NASH through ferroptosis and serve as molecular diagnostic markers and drug targets.

However, several restrictions of the present research should be addressed. 1) Bioinformatics results were obtained from analysis of liver tissue from NASH patients and healthy individuals in public databases, which were supposed to be prospectively verified in subsequent clinical trials. 2) Our results are limited to investigating the change of gene expression level, while the biological function of the hub genes using loss-of-function and gain-of-function assays has not been explored, which is the main purpose of our next research. 3) The FRGs were generated from FerrDb, and the website is constantly updated.

## 5 Conclusion

In this study, we uncovered the ferroptosis-related genes functioning in NASH using bioinformatics and a ferroptosis database based on literature. Our data indicated that a dramatic correlation exists between the fibrosis stage of NASH and the expression of the ferroptosis-related genes *CDKN1A* and *SIRT1*. Additionally, the diagnostic model based on *CDKN1A* and *SIRT1* has a remarkable capacity to diagnose NASH. Overall, our findings provide novel perspectives and therapeutic targets for future research on ferroptosis in NASH.

## Data availability statement

The datasets presented in this study can be found in online repositories. The names of the repository/repositories and accession number(s) can be found in the article/**Supplementary Material**.

## Ethics statement

The animal study was reviewed and approved by the Ethics Committee of Chongqing Medical University.

## Author contributions

LH conceived and designed the investigation. LH and JW performed the experiment and wrote the original draft. BT and RZ analyzed and visualized the results, and revised the manuscript. CL and BN supervised the investigation and provided pivotal suggestions on the final version of the manuscript. All authors contributed to the article and approved the submitted version.
