# Supplementary material for: Identification of ferroptosis-related genes in the progress of NASH
Source: Front Endocrinol (Lausanne). 2023 May 25;14:1184280. doi: 10.3389/fendo.2023.1184280 (PMC10247994; doi:10.3389/fendo.2023.1184280)
Supplement: Supplementary file 1 [file Table_1.docx]

Supplementary Material

Supplementary Table S1 GO terms enriched by 42 candidate genes

| GO ID | GO Term | Number  of Genes | P Value |
| --- | --- | --- | --- |
| GO:0000096 | sulfur amino acid metabolic process | 10 | 8.01E-05 |
| GO:0001676 | long-chain fatty acid metabolic process | 10 | 2.17E-07 |
| GO:0002673 | regulation of acute inflammatory response | 8 | 2.05E-04 |
| GO:0002675 | positive regulation of acute inflammatory response | 6 | 4.32E-05 |
| GO:0006633 | fatty acid biosynthetic process | 5 | 3.08E-12 |
| GO:0006925 | inflammatory cell apoptotic process | 5 | 2.23E-05 |
| GO:0007566 | embryo implantation | 5 | 3.14E-04 |
| GO:0008207 | C21-steroid hormone metabolic process | 4 | 8.67E-05 |
| GO:0010565 | regulation of cellular ketone metabolic process | 4 | 6.21E-10 |
| GO:0010574 | regulation of vascular endothelial growth factor production | 4 | 5.22E-05 |
| GO:0010575 | positive regulation of vascular endothelial growth factor production | 4 | 3.52E-05 |
| GO:0010883 | regulation of lipid storage | 3 | 2.30E-04 |
| GO:0019217 | regulation of fatty acid metabolic process | 3 | 2.27E-06 |
| GO:0019369 | arachidonic acid metabolic process | 3 | 3.14E-04 |
| GO:0019915 | lipid storage | 3 | 4.18E-05 |
| GO:0032722 | positive regulation of chemokine production | 3 | 2.47E-05 |
| GO:0033028 | myeloid cell apoptotic process | 3 | 1.52E-04 |
| GO:0033559 | unsaturated fatty acid metabolic process | 3 | 1.33E-13 |
| GO:0035633 | maintenance of blood-brain barrier | 3 | 8.01E-05 |
| GO:0036109 | alpha-linolenic acid metabolic process | 3 | 3.59E-06 |
| GO:0042304 | regulation of fatty acid biosynthetic process | 3 | 5.85E-08 |
| GO:0042448 | progesterone metabolic process | 3 | 7.99E-06 |
| GO:0043651 | linoleic acid metabolic process | 3 | 1.76E-07 |
| GO:0045453 | bone resorption | 3 | 5.38E-04 |
| GO:0045600 | positive regulation of fat cell differentiation | 3 | 5.60E-04 |
| GO:0045723 | positive regulation of fatty acid biosynthetic process | 3 | 1.72E-05 |
| GO:0045923 | positive regulation of fatty acid metabolic process | 3 | 8.67E-05 |
| GO:0046626 | regulation of insulin receptor signaling pathway | 3 | 4.35E-04 |
| GO:0046686 | response to cadmium ion | 3 | 5.16E-04 |
| GO:0050873 | brown fat cell differentiation | 3 | 7.45E-06 |
| GO:0070498 | interleukin-1-mediated signaling pathway | 3 | 3.16E-05 |
| GO:0070542 | response to fatty acid | 3 | 4.45E-07 |
| GO:0071398 | cellular response to fatty acid | 3 | 7.39E-05 |
| GO:0090335 | regulation of brown fat cell differentiation | 3 | 1.96E-05 |
| GO:0150076 | neuroinflammatory response | 3 | 5.38E-04 |
| GO:0150077 | regulation of neuroinflammatory response | 3 | 6.80E-05 |
| GO:1900076 | regulation of cellular response to insulin stimulus | 3 | 4.95E-04 |
| GO:1901661 | quinone metabolic process | 3 | 9.36E-05 |
| GO:1903202 | negative regulation of oxidative stress-induced cell death | 3 | 2.56E-04 |
